# Relationship between CMR and ECG-derived indices of left ventricular hypertrophy

**DOI:** 10.1186/1532-429X-11-S1-P289

**Published:** 2009-01-28

**Authors:** Richard M Nethononda, Barbara Casadei, Polly Whitworth, Jane Francis, Nicola Alder, Hugh Watkins, Stefan Neubauer

**Affiliations:** University of Oxford, Oxford, UK

**Keywords:** Left Ventricular Hypertrophy, Interstitial Component, Cornell Voltage, Voltage Index, Total Myocyte

## Background

Left ventricular hypertrophy (LVH) is associated with increased risk of cardiovascular events. ECG is the most commonly used technique for detecting LVH. CMR is the gold standard for measurement of cardiac parameters including LV mass. The objective of this study was therefore to investigate the relationship between ECG- and CMR-derived indices of LVH in a cohort with substantial prevalence of LVH.

## Methods

ECG and CMR data of subjects from the Oxford Family Blood Pressure cohort was evaluated. An ECG was obtained from each subject. QRS duration was measured from the beginning of the Q wave to the end of the S wave. Sokolow-Lyon voltage was defined as SV1+RV5. Cornell voltage was defined as RaVL+SV3. The 12-lead sum voltage was the sum of QRS amplitudes in all 12 leads. These voltage indices were multiplies by the QRS duration as an expression of their time voltage index. ECG-derived LV mass was calculated according to the methods of Siegel. Short-axis end-expiration cine images of the left ventricle were acquired from each subject using a 1.5 T scanner and analysed. Categorical and continuous variables were compared using Chi-squared and Student's t-test, respectively. Pearson's correlation was used to evaluate the strength of the relationship between ECG and CMR parameters. The Bland-Altman method was used to evaluate agreement between LVM measured by both ECG and CMR. *P* value < 0.05 was considered significant.

## Results

There were 152 subjects, 83 females. Hypertension was more prevalent, and ambulatory BP as well as waist-to-hip ratios was higher amongst males. QRS duration, Sokolow-Lyon, 12-lead sum of QRS voltage and ECG-derived LVM were significantly larger in males than in females. Males had larger left ventricular mass and wall thickness. In males, there was a significant correlation between CMR LV mass and ECG indices such as QRS duration, 12-lead sum duration, Sokolow-Lyon duration and Cornell duration (Table 1). QRS duration demonstrated the highest correlation with wall thickness, whilst both the 12-lead sum and Cornell voltages demonstrated marginally significant correlation with this parameter. In contrast, females (Table 2) showed no significant correlations between CMR LV mass and ECG indices such as QRS duration, 12 lead sum of voltages and Sokolow-Lyon voltages. Only Cornell voltage showed a marginally significant correlation with CMR LVM. Similarly, both QRS duration and 12-lead sum of QRS voltages showed no significant correlations with LV wall thickness. There was only marginally significant correlation between LV wall thickness and both the Sokolow-Lyon and Cornell voltage indices. The 95% limits of agreement between LV mass measured by ECG and by CMR were -53.3 g to 54.7 g indicating that ECG overestimates LVM in those with LVM in the lower ranges and underestimates it in those with LVM in the upper ranges.

**Table 1:** Correlation coefficients (r) between ECG and CMR parameters in males

	Total edwt (mm)	Total eswt (mm)	CMR LVM (g)
QRS duration (msec)	0.32**	0.46***	0.49***
SL voltage duration (mV/msec)	0.13	0.24*	0.35**
Cornell duration (mV/msec)	0.26*	0.27*	0.37**
12-lead sum duration (mV/msec)	0.26*	0.35**	0.43***
ECG LVM (g)	0.40**	0.44***	0.62***

edwt and eswt, the sum of end-diastolic and end-systolic wall thickness; CMR, cardiac magnetic resonance; LVM/I, left ventricular mass and mass index; SL, Sokolow-Lyon. *p < 0.05, **p < 0.01 and ***p < 0.001.

**Table 2:** Correlation coefficients (r) of ECG and CMR variables in females

	Total edwt (mm)	Total eswt (mm)	CMR LVM(g)
QRS duration (msec)	0.09	0.16	0.21
SL voltage duration (mV/msec)	0.19	0.30**	0.21
Cornell duration (mV/msec)	0.22*	0.27*	0.29*
12-lead sum duration (mV/msec)	0.13	0.13	0.18
ECG LVM (g)	0.25*	0.34**	0.35**

edwt and eswt, end-diastolic and systolic wall thickness; CMR, cardiac magnetic resonance; LVM, left ventricular mass; SL, Sokolow-Lyon. *p < 0.05 and **p < 0.01.

## Conclusion

The lower correlations in indices of left ventricular hypertrophy measured using the two techniques possibly reflect the different components of, and physiological processes within myocardium that they each measure. ECG measures electrical activity within myocardium, whilst CMR measures total myocyte mass, non myocyte cells and interstitial components. The difference in chest geometry between genders is a possible explanation for the poor correlations of ECG vs. CMR indices of left ventricular hypertrophy in females compared to male subjects.

